# MicroRNA-16, via FGF2 Regulation of the ERK/MAPK Pathway, Is Involved in the Magnesium-Promoted Osteogenic Differentiation of Mesenchymal Stem Cells

**DOI:** 10.1155/2020/3894926

**Published:** 2020-04-27

**Authors:** Hong Qi, Yang Liu, Lu Wu, Su Ni, Jing Sun, Junchao Xue, Qizhan Liu, Xinye Ni, Weimin Fan

**Affiliations:** ^1^Center for Global Health, The Key Laboratory of Modern Toxicology, Ministry of Education, School of Public Health, Nanjing Medical University, Nanjing, 211166 Jiangsu, China; ^2^Department of Orthopedics, The First Affiliated Hospital, Nanjing Medical University, Nanjing, 210029 Jiangsu, China; ^3^Second People's Hospital of Changzhou, Nanjing Medical University, Changzhou, 213003 Jiangsu, China

## Abstract

microRNAs (miRNAs) participate in the osteogenic differentiation of bone marrow mesenchymal stem cells (BMSCs). However, few reports have discussed the effect of miRNAs on the magnesium chloride (MgCl_2_)-induced promotion of osteogenic differentiation of BMSCs, a process involved in the healing of bone tissue. As determined in the present investigation, MgCl_2_ decreased miR-16 levels; increased levels of fibroblast growth factor 2 (FGF2), p-p38, and p-ERK; and promoted the osteogenic differentiation of BMSCs. Enhancement of miR-16 levels by an miR-16 mimic blocked these MgCl_2_-induced changes. Moreover, luciferase reporter assays confirmed that miR-16 binds to the 3′UTR region of *FGF2* mRNA. Down-regulation of FGF2 blocked the MgCl_2_-induced increases of p-p38 and p-ERK and the promotion of the osteogenic differentiation of BMSCs. Furthermore, over-expression of miR-16 attenuated the MgCl_2_-induced overproduction of p-p38 and p-ERK1/2 and the high levels of osteogenic differentiation, effects that were reversed by elevated expression of FGF2. In summary, the present findings provide a mechanism by which miR-16 regulates MgCl_2_-induced promotion of osteogenic differentiation by targeting FGF2-mediated activation of the ERK/MAPK pathway.

## 1. Introduction

Magnesium (Mg) is an essential element in human physiology; in the body, bone stores 67% of all Mg [1]. Mg ions are involved in various metabolic processes, particularly mineral metabolism, in which they promote calcification of bone cells [2]. For various species, a deficiency of Mg ions leads to osteoporosis due to decreased bone formation and increased bone resorption [3]. To maintain proper physiological function, the amount of Mg in bone is regulated dynamically by skeletal remodeling during bone resorption and formation [1]. Mg promotes bone formation through activation of Notch signaling and Wnt/*β*-catenin pathway [4, 5]. Calcitonin gene-related peptide (CGRP) is involved in Mg-induced enhancement of bone-fracture healing [6]. However, the mechanisms by which Mg ions regulate bone repair remain unclear.

Bone, a metabolically active tissue, is continuously remodeled during development and throughout life to repair micro-damage; bone adjusts its architecture to changing mechanical needs [7]. This dynamic process relies on the coordinated and timely balance between bone resorption by osteoclasts and bone formation by osteoblasts. Osteoblasts arise from bone marrow mesenchymal stem cells (BMSCs), which are rare, pluripotent cells that, in response to specific stimuli from the microenvironment, activate the genetic program leading to osteoblast formation [8]. There is a growing interest in BMSCs because of their use in cell-based therapy as a strategy in orthopedics. It is therefore essential to identify the molecular events involved in their differentiation into osteoblasts. Both chemical and physical cues modulate the fate commitment of BMSCs [9]. As potential biomaterials for orthopedic implants, biodegradable Mg-containing materials have advantages, including self-degradation and promotion of bone repair [10].

Fibroblast growth factor 2 (FGF2), which often localizes to the nucleus and/or to cytoplasm, is a mitogen for bone-derived cells [11]. Systemic and local administration of FGFs increases bone formation and accelerates callus remodeling and healing of fractures [12–14]. Initial dosing with FGF2 could increase the pool of committed progenitor cells, and continuous FGF2 might block later phases of osteoblast differentiation, which appear to be regulated by other growth factors such as bone morphogenetic protein 2 [15, 16]. Runx2 is a transcription factor specific for expression of genes involved in the differentiation and development of osteoblasts, such as *ALP*, *COL I*, and *OCN*, and in activation of the Ras/MAPK/ERK pathway in osteogenesis [17]. Moreover, FGF2 activates ERK and p38 MAPK migration into the nucleus, which triggers the activation of nuclear transcription factors, thereby leading to downstream gene expression [18].

microRNAs (miRNAs) are a family of highly conserved, short non-coding RNAs that regulate gene expression by base pairing with the 3′-untranslated region (3′-UTR) to enhance mRNA degradation or inhibit post-transcriptional translation [19]. In addition, miRNAs are negative regulators of diverse biological and pathological processes, including developmental timing, organogenesis, apoptosis, cell proliferation, and differentiation and control of tumorigenesis [20–22]. In the osteogenic differentiation of BMSCs, miRNAs have an essential role [23]. For instance, microRNA-30e inhibits the differentiation of osteoprogenitors by targeting low-density lipoprotein receptor-related protein 6 [24]. In BMSCs, miRNA-21 promotes osteogenesis via the PTEN/PI3K/Akt pathway, and, for animals, *β*-tricalcium phosphate scaffolds seeded with miRNA-21-modified BMSCs enhance new bone formation in critical size defects [25]. The role of miRNAs in Mg-induced promotion of osteogenic differentiation of BMSCs deserves further research.

In the present study, we demonstrated that, by targeting FGF2 in BMSCs, downregulation of miR-16 contributed to the promotion of osteoblast differentiation of BMSCs via the ERK/MAPK pathway induced by MgCl_2_. Such information contributes to an understanding of the mechanisms by which MgCl_2_ promotes bone regeneration.

## 2. Materials and Methods

### 2.1. Isolation and Culture of BMSCs

Animal procedures were approved by the Institutional Animal Care and Use Committee (IACUC) of Nanjing Medical University. Male 3-week-old Sprague-Dawley rats were purchased from the Experimental Animal Center of Nanjing Medical University. Primary BMSCs were isolated and characterized as described previously [26, 27]. Under sterile conditions, BMSCs were collected by flushing the femurs and tibias with alpha minimal essential medium (*α*MEM; Sigma-Aldrich CO, St. Louis, MO, USA) containing fetal bovine serum (FBS, 15%; Lonza Inc., Walkersville, MD, USA), ultraglutamine (1%, Lonza), penicillin (100 U/mL), and streptomycin (100 *μ*g/mL). The cells were cultured at 37°C, with 5% CO_2_, in a humidified incubator. The BMSCs culture medium was replaced every other day.

### 2.2. Osteogenic Differentiation

For induction of osteogenesis, BMSCs were seeded in 6-well or 24-well plates. When they reached a density of 80%–90%, the BMSCs were grown in DMEM-HG medium supplemented with 10% FBS, 10 nmol/L dexamethasone (Sigma Aldrich, USA), 10 mmol/L *β*-glycerol phosphate (Sigma Aldrich, USA), 50 *μ*g/mL ascorbic acid (Sigma Aldrich, USA), 1% L-glucose, 1% penicillin-streptomycin, and 1% HEPES. The osteogenic induction medium was replaced every three days.

### 2.3. MgCl_2_ Treatment

For osteogenic differentiation of BMSCs, MgCl_2_ (Sigma Aldrich, USA) at concentrations of 0, 2.5, or 5.0 mM (excluding the concentration of Mg ions in the culture medium) was added into the osteogenic induction medium to replace the growth medium when BMSCs seeded in the culture plates reached 60% confluence.

### 2.4. Cell Viability Assay

Cell viability was assessed with 3-(4,5-dimethylthiazol-2-yl)-2,5-diphenyltetrazolium bromide (MTT) (Sigma Aldrich, USA) following the manufacturer's instructions. BMSCs (1 × 10^4^ cells per well) were seeded in 96-well plates. After 24 h, they were exposed to 0, 2.5, 5.0 10, 25, 50, or 100 mM MgCl_2_ (Sigma Aldrich, USA) for 24 h or for 7 days. After treatment, the MTT reagent, diluted to a concentration of 0.5 mg/ml with osteogenesis-inducing medium, was added to the plates, which were incubated at 37°C for 4 h. Next, the MTT was replaced by dimethyl sulfoxide (Sigma Aldrich, USA), the preparations were incubated at 37°C for another 15 min, and then the plates were agitated softly for 15 min. The absorbance of each well was recorded at 490 nm by an Infinite M200 Pro instrument (TECAN, Switzerland). Each assay was repeated at least three times independently.

### 2.5. Cell Transfection

An miR-16 mimic, miR-16 inhibitor, miRNA negative control mimic (con mimic), miRNA negative control inhibitor (con inhibitor), sh-FGF2, control shRNA, pGC-LV- FGF2-GFP (LV- FGF2), and GC-LV- FGF2-Control-GFP (LV-NC) were synthesized by Genechem (Shanghai, China). Cells were transiently transfected according to the manufacturer's protocol. After transfection, cells were harvested and used for experiments.

### 2.6. Luciferase Reporter Analysis

To investigate the effect of miR-16 on the 3'UTR of FGF2 (FGF2-3'UTR), the 3'UTR sequence of FGF2, which was predicted to harbor the miR-16 seed region (AUGACGAU), or a mutant sequence (TGCTGCTA) was inserted into the XhoI and NotI sites of the psiCHECK-2 promoter vector (GENEray, China). These were named psiCH-FGF2-wt and psiCH- FGF2-mut, respectively. For the reporter assays, psiCH- FGF2-wt or psiCH- FGF2-mut were co-transfected into the cells with an miR-16 mimic or a negative control mimic. After 48 h of transfection, the cells were harvested for detection using the Dual Luciferase Reporter Assay system (Promega, USA) with an Infinite M200 PRO multimode microplate reader (TECAN, Swiss). Renilla luciferase activities were used to normalize the transfection efficiency.

### 2.7. Alkaline Phosphatase (ALP) Assay

ALP activity was determined with Sensolyte® pNPP Alkaline Phosphatase Assay Kits (Anaspec, USA) according to the manufacturer's instructions. BMSCs were seeded into 96-well plates at a density of 1 × 10^4^ cells/well. At 24 h after plating, BMSCs were exposed to MgCl_2_ with osteogenic differentiation for 7 days. Cells were washed twice with assay buffer, lysed with Triton-X-100, and collected in microcentrifuge tubes. After incubation at 4°C for 10 min under agitation, the cells were centrifuged at 2500 g for 10 min to collect the supernatant. The supernatant was incubated with p-nitrophenyl phosphate substrate solution, and the absorbance was read at 405 nm with an Infinite M200 Pro (TECAN, Switzerland) instrument. The ALP activity was normalized against protein concentration measured with BCA Protein Assay Kits (Beyotime Institute of Biotechnology, China). For each test, three samples were used.

### 2.8. ALP Staining

Leukocyte Alkaline Phosphatase Kits (Sigma Aldrich, USA) were used for ALP staining according to the manufacturer's instructions. BMSCs were seeded in 24-well plates at a density of 7 × 10^4^ cells/well in growth culture medium. When their confluence reached 60%, BMSCs were exposed to MgCl_2_ with osteogenic differentiation for 14 days. After that, cells were fixed with 4% formaldehyde and 5% citrate in acetone at room temperature for 30 s. The fixed cells were washed with PBS and incubated with 0.2% naphthol AS-BI and 0.2% diazonium salt at room temperature for another 15 min. After washing the plates with PBS, images were taken at 10× magnification under an optical microscope (Nikon, Japan).

### 2.9. Alizarin Red S Staining

MSCs were seeded into 24-well plates at a density of 7 × 10^4^ cells/well and were exposed to MgCl_2_ with osteogenic differentiation for 14 days. The cells were washed with PBS, fixed with 10% formaldehyde at room temperature for 10 min, and incubated with 40 mM alizarin red S (Sigma Aldrich, USA) solution at room temperature for 20 min. After discarding the solutions and washing the plates with PBS 4 times, images were made at 10× magnification under an optical microscope (Nikon, Japan).

### 2.10. RNA Preparation and Quantitative Real-Time Polymerase Chain Reaction (qRT-PCR)

Total RNA was isolated from cells with TRIzol reagent (Invitrogen Life Technologies Co, USA) according to manufacturer's protocol. The purity and concentration of total RNA was assessed with a NanoDrop 2000 (Thermo Fisher Scientific, USA). Reverse transcription was accomplished using Prime Script™ RT Reagent Kits with gDNA Eraser (Perfect Real Time, Takara, Japan) with 1 *μ*g of RNA according to the manufacturer's instructions. qRT-PCR was performed with an ABI7900 Fast Real-Time System (Applied Bio systems, USA) using SYBR Premix Ex Taq™ Kits (Takara, Japan). Glyceraldehyde 3-phosphate dehydrogenase (GAPDH) was used as an internal standard, and the relative expressions of genes were calculated by the 2^-*Δ*ΔCt^ method [28]. For each test, three samples were used. Primer sequences are shown in Table 1.

### 2.11. Western Blots

Cell were lysed with RIPA (Beyotime Institute of Biotechnology, China) according to manufacturer's protocol, and protein concentrations were quantified with BCA Protein Assay kits (Beyotime Institute of Biotechnology, China). Equal amounts (50 *μ*g) of protein were separated by 10% sodium dodecyl sulfate-polyacrylamide gel electrophoresis and were transferred to polyvinylidene fluoride membranes (Millipore, Billerica, MA). Membranes were incubated overnight at 4°C with a 1 : 1000 dilution of anti-GAPDH (Beyotime, China) and an antibody for ALP (Abcam, USA), Runx2 (Cell Signaling Technology, USA), Sp7 (Abcam, USA), OCN (Abcam, USA), OPN (Abcam, USA), FGF2 (Cell Signaling Technology, USA), p38 (Cell Signaling Technology, USA), p-p38 (Cell Signaling Technology, USA), ERK1/2 (Abcam, USA), or p-ERK1/2 (Abcam, USA). After additional incubation with a 1 : 1000 dilution of HRP-conjugated goat anti-mouse and goat anti-rabbit secondary antibodies (Jackson ImmunoResearch, USA) for 1 h, the immune complexes were detected by enhanced chemiluminescence (Cell Signaling Technology, USA). The intensities of bands were quantified with ImageJ software.

### 2.12. Statistical Analyses

All data values were expressed as means ± standard deviations (SD). Graphpad 7.0 was applied for statistical analyses. One-way analysis of variance (ANOVA) was used for comparisons of means among multiple groups, and a multiple-range least significant difference (LSD) was used for inter-group comparisons. All statistical analyses were performed with SPSS 19.2, and data were marked with (^∗^) for *p* < 0.05.

## 3. Results

### 3.1. Cytotoxicity of MgCl_2_ to BMSCs

To assess the cytotoxicity of MgCl_2_, tests using MTT were conducted. The results showed that, after treatment of BMSCs with MgCl_2_ for 24 h, 7 days or 14 days, concentrations of ≤25 mM, ≤10 mM and ≤ 5 mM, respectively, had no cytotoxicity to BMSCs. At concentrations of ≥50 mM (for 24 h), ≥25 mM (for 7 days), or ≥ 10 mM (for 14 days), cell viability was decreased in a concentration-dependent manner (Figure S1A-C). Therefore, we chose concentrations of 2.5 and 5.0 mM MgCl_2_ to treat BMSCs in the following studies.

### 3.2. MgCl_2_ Promotes the Osteogenic Differentiation of BMSCs

**Figure 1:**
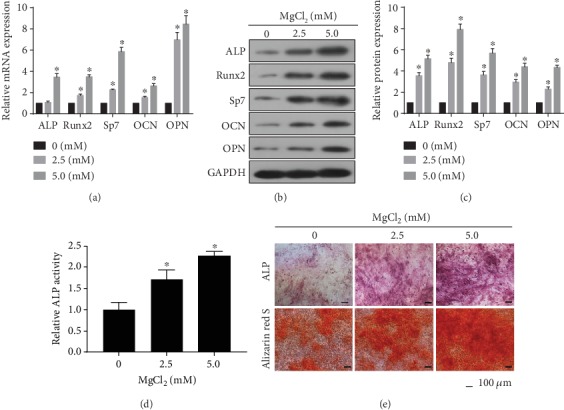
MgCl_2_ promotes the osteogenic differentiation of BMSCs. MSCs were exposed to 0, 2.5, or 5.0 mM MgCl_2_ and subjected to osteogenic differentiation for 7 days. (a) The mRNA levels of alkaline phosphatase (*ALP*), *Runx2*, osterix (*Sp7*), osteocalcin (*OCN*), and osteopontin (*OPN*) were measured by qRT-PCR (mean ± SD, *n* = 3). ^∗^ *p* < 0.05, different from BMSCs in the absence of MgCl_2_. (b) Western blots were performed, and (c) relative protein levels of ALP, Runx2, Sp7, OCN, and OPN were determined (mean ± SD, *n* = 3). ^∗^ *p* < 0.05, different from BMSCs in the absence of MgCl_2_. BMSCs were exposed to 0, 2.5, or 5.0 mM MgCl_2_ and subjected to osteogenic differentiation for 10 days. (d) ALP activity was detected by ALP assays (mean ± SD, *n* = 3). ^∗^ *p* < 0.05, different from BMSCs in the absence of MgCl_2_. BMSCs were exposed to 0, 2.5, or 5.0 mM MgCl_2_ and subjected to osteogenic differentiation for 14 days. (e) The ALP content and the numbers of mineralization nodules were evaluated by ALP staining (upper) and alizarin red S staining (lower). Scale bar, 100 *μ*m.

Mg-containing substances have emerged as components of a new class of biodegradable biomaterials for tissue engineering and medical devices to avoid implant removal and to circumvent long-term effects of non-degradable, permanent implants. Mg-containing materials exhibit advantages, especially for load-bearing orthopedic and cardiovascular devices [29–33]. Mg ions are involved in various biological functions, including bone and mineral homeostasis [34]. In the present study, BMSCs were exposed to 0, 2.5, or 5.0 mM MgCl_2_ for 7, 10, or 14 days. MgCl_2_ increased the mRNA expressions of the osteogenic master genes alkaline phosphatase (*ALP*), runt-related transcription factor 2 (*Runx2*), osterix (*Sp7*), osteocalcin (*OCN*), and osteopontin (*OPN*) (Figure 1(a)); their protein levels were also enhanced (Figure 1(b)-1(c)). For BMSCs differentiated into osteoblasts with 2.5 or 5.0 mM MgCl_2,_ ALP activity increased (Figure 1(d)). Likewise, ALP content, verified by ALP staining, and matrix mineralization, verified by Alizarin Red S, were more abundant in cells cultured with 2.5 or 5.0 mM MgCl_2_, in which there was a dose-effect relationship (Figure 1(e) and Figure S2). These results suggested that MgCl_2_ promotes the osteogenic differentiation of BMSCs.

### 3.3. MgCl_2_ Causes the Decreases of miR-16 Levels, the Increases of FGF2 Levels, and the Activation of ERK/MAPK Pathway

**Figure 2:**
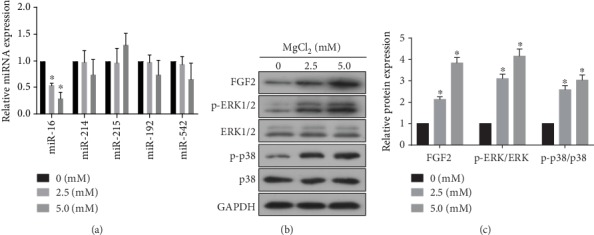
MgCl_2_ causes the decreases of miR-16 levels, the increases of FGF2 levels, and the activation of ERK/MAPK pathway. BMSCs were exposed to 0, 2.5 or 5.0 mM MgCl_2_ for 24 h. (a) The levels of miR-16, miR-214, miR-215, miR-192, and miR-542 were determined by qRT-PCR (mean ± SD, *n* = 3). ^∗^ *p* < 0.05, different from BMSCs in the absence of MgCl_2_. (b) Western blots were performed, and (c) relative protein levels of FGF2, p-ERK1/2, ERK1/2, p-p38, and p38 were determined (mean ± SD, *n* = 3). ^∗^ *p* < 0.05, different from BMSCs in the absence of MgCl_2_.

FGF2, which activates Runx2 by phosphorylation through the Ras/MAPK/ERK pathway, is involved in osteogenesis [17]. FGF2 is a target of miR-16 [35]. To determine if miR-16, miR-214, miR-215, miR-192, miR-542, FGF2, and the ERK/MAPK pathway are involved in the effects of MgCl_2_ on BMSCs, BMSCs were exposed to 2.5 or 5.0 mM MgCl_2_ for 24 h. MgCl_2_ decreased the levels of miR-16 (Figure 2(a)); however, the expression of the other miRNAs did not change appreciably. Thus, we chose miR-16 for further research. Moreover, the protein levels of FGF2, p-p38, and p-ERK1/2 were elevated after 24 h of treatment with MgCl_2_ (Figure 2(b)–2(c)). These results suggested that MgCl_2_ caused the decreases of miR-16 levels, the increases of FGF2 levels, and the activation of ERK/MAPK pathway, which may be related to the promotion of osteogenic differentiation of BMSCs induced by MgCl_2_.

### 3.4. FGF2 Is Involved in MgCl_2_-Induced Activation of the ERK/MAPK Pathway

**Figure 3:**
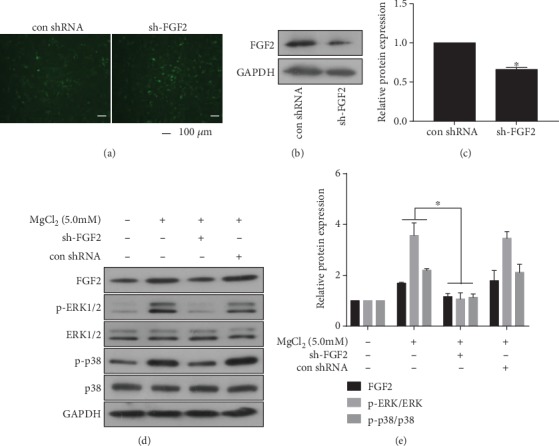
FGF2 is involved in MgCl_2_-induced activation of the ERK/MAPK pathway. BMSCs were transfected with sh-FGF2 or control-shRNA for 24 h. (a) The fluorescence of BMSCs after transfection with sh-FGF2 or control-shRNA. (b) Western blots were performed, and (c) relative protein levels of FGF2 were determined (mean ± SD, *n* = 3). ^∗^ *p* < 0.05, different from control-shRNA BMSCs. BMSCs were transfected with sh-FGF2 or control-shRNA for 24 h, then exposed to 0 or 5.0 mM MgCl_2_ for 48 h. (d) Western blots were performed, and (e) relative protein levels of FGF2, p-ERK1/2, ERK1/2, p-p38, and p38 were determined (mean ± SD, *n* = 3). ^∗^ *p* < 0.05, different from control-shRNA BMSCs.

To determine the effects of FGF2 on the ERK/MAPK pathway in BMSCs, we constructed sh-FGF2 and established its transfection efficacy (Figure 3(a)). The expression of FGF2 was decreased (Figure 3(b)-3(c)). Next, we found that, after BMSCs (treated with MgCl_2_) were transfected with sh-FGF2, the higher levels of FGF2, p-p38, and p-ERK1/2 were not evident (Figure 3(d)-3(e)). Thus, for BMSCs, FGF2 was involved in the MgCl_2_-induced activation of ERK/MAPK pathway.

### 3.5. FGF2 Is Involved in MgCl_2_-Induced Promotion of Osteogenic Differentiation of BMSCs

**Figure 4:**
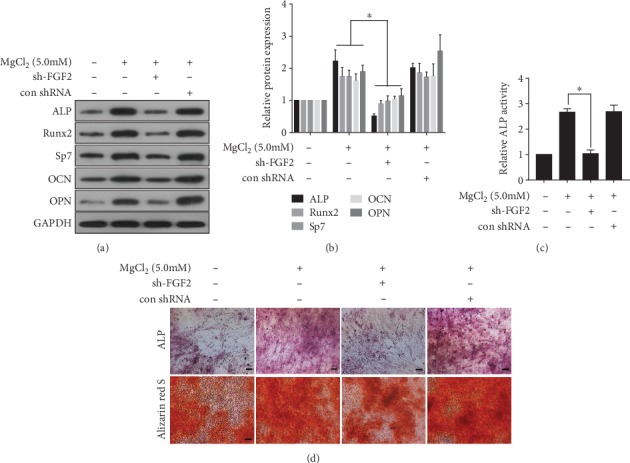
FGF2 is involved in MgCl_2_-induced inhibition of osteogenic differentiation of BMSCs. BMSCs were transfected with sh-FGF2 or control-shRNA for 24 h, then exposed to 0 or 5.0 mM MgCl_2_ and subjected to osteogenic differentiation for 7 days. (a) Western blots were performed, and (b) relative protein levels of ALP, Runx2, Sp7, OCN, and OPN were determined (mean ± SD, *n* = 3). ^∗^ *p* < 0.05, different from control-shRNA BMSCs treated with MgCl_2_. BMSCs were transfected with sh-FGF2 or control-shRNA for 24 h, then exposed to 0 or 5.0 mM MgCl_2_ and subjected to osteogenic differentiation for 10 days. (c) ALP activity was measured by ALP assays (mean ± SD, *n* = 3). ^∗^ *p* < 0.05, different from control-shRNA BMSCs treated with MgCl_2_. BMSCs were transfected with sh-FGF2 or control-shRNA for 24 h, then exposed to 0 or 5.0 mM MgCl_2_ and subjected to osteogenic differentiation for 14 days. (d) The ALP content and the numbers of mineralization nodules were evaluated by ALP staining (upper) and alizarin red S staining (lower). Scale bar, 100 *μ*m.

We hypothesized that FGF2 promoted osteogenic differentiation of BMSCs via regulation of the ERK/MAPK pathway. After BMSCs (treated with MgCl_2_) were transfected with sh-FGF2, high levels of ALP, RUNX2, Sp7, OCN, and OPN were not evident (Figure 4(a)-4(b)), and ALP activity was lower (Figure 4(c)). Moreover, FGF2 down-expression reduced the extensive ALP staining and alizarin red S staining seen after MgCl_2_ exposure (Figure 4(d)). Thus, FGF2 participated in MgCl_2_-induced promotion of osteogenic differentiation of BMSCs.

### 3.6. miR-16 Is Involved in the MgCl_2_-Induced Increase of FGF2 and Activation of the ERK/MAPK Pathway

**Figure 5:**
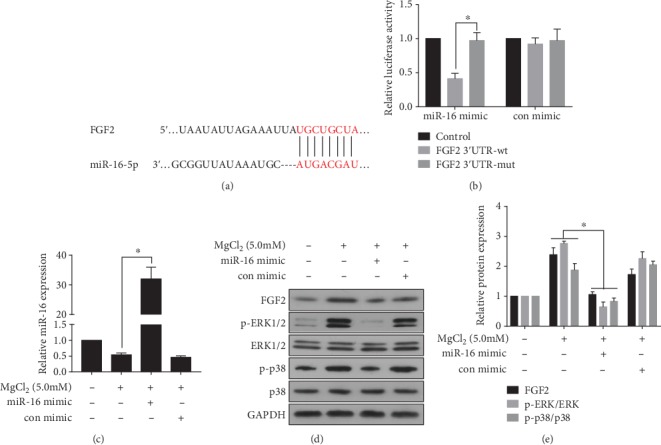
miR-16 is involved in the MgCl_2_-induced increase of FGF2 and activation of the ERK/MAPK pathway. (a) Schematic graph illustrating binding sites between miR-16 and the 3′UTR region of FGF2. BMSCs were co-transfected for 24 h with the miR-16 mimic or negative control and FGF2-wt plasmid or FGF2-mut plasmid. (b) Luciferase activity was measured. ^∗^*p* < 0.05, different from BMSCs in the absence of the miR-16 mimic. BMSCs were transfected with the miR-16 mimic or a negative control for 24 h, then exposed to 0 or 5.0 mM MgCl_2_ for 48 h. (c) The levels of miR-16 were determined by qRT-PCR (mean ± (a). SD, *n* = 3). ^∗^ *p* < 0.05, different from BMSCs treated with MgCl_2_ in the absence of the miR-16 mimic. (D) Western blots were performed, and (E) relative protein levels of FGF2, p-ERK1/2, ERK1/2, p-p38, and p38 were determined (mean ± SD, *n* = 3). ^∗^ *p* < 0.05, different from BMSCs treated with MgCl_2_ in the absence of the miR-16 mimic.

The bioinformatics tool TargetScan (http://www.targrtscan.org/) was used to predict the binding sites for miR-16 within the 3'UTR region of *FGF2* (Figure 5(a)). Luciferase assays revealed that transfection of cells with the miR-16 mimic inhibited the luciferase activity of the *FGF2* 3'UTR, but the *FGF2* mutant showed no response to the mimic (Figure 5(b)), indicating that, for BMSCs, miR-16 binds to the 3′UTR of *FGF2.* To determine the interaction of miR-16 and MgCl_2_ in BMSCs, miR-16 was elevated by transfection with the miR-16 mimic (Figure 5(c)). Ectopic expression of miR-16 attenuated the MgCl_2_-induced up-regulation of FGF2, p-p38, and p-ERK1/2 (Figure 5(d)-5(e)). These results suggested that miR-16 is involved in the MgCl_2_-induced activation of the ERK/MAPK pathway.

### 3.7. miR-16 Is Involved in MgCl_2_-Induced Promotion of Osteogenic Differentiation of BMSCs

**Figure 6:**
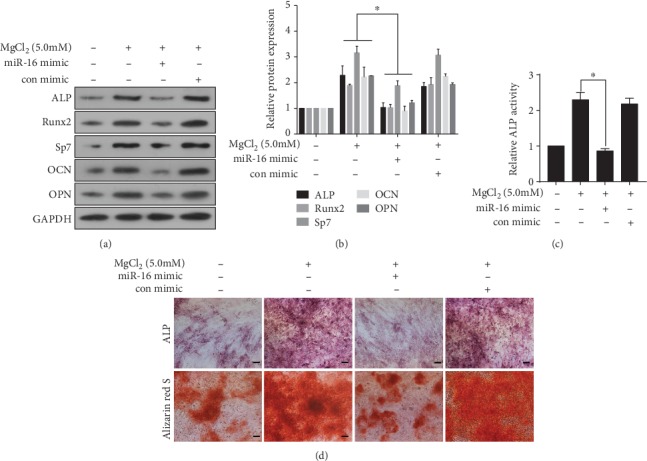
miR-16 is involved in MgCl_2_-induced inhibition of osteogenic differentiation of BMSCs. BMSCs were transfected with miR-16 mimic or negative control for 24 h, then exposed to 0 or 5.0 mM MgCl_2_ and subjected to osteogenic differentiation for 7 days. (a) Western blots were performed, and (b) relative protein levels of ALP, Runx2, Sp7, OCN, and OPN were determined (mean ± SD, *n* = 3). ^∗^ *p* < 0.05, different from BMSCs treated with MgCl_2_ in the absence of the miR-16 mimic. BMSCs were transfected with the miR-16 mimic or negative control for 24 h, then exposed to 0 or 5.0 mM MgCl_2_ and subjected to osteogenic differentiation for 10 days. (c) ALP activity was detected by ALP assays (mean ± SD, *n* = 3). ^∗^ *p* < 0.05, different from BMSCs treated with MgCl_2_ in the absence of the miR-16 mimic. BMSCs were transfected with miR-16 mimic or negative control for 24 h, then exposed to 0 or 5.0 mM MgCl_2_ and subjected to osteogenic differentiation for 14 days. (d) The ALP content and the numbers of mineralization nodules were evaluated by ALP staining (upper) and alizarin red S staining (lower). Scale bar, 100 *μ*m.

To determine if miR-16 is involved in the MgCl_2_-induced promotion of osteogenic differentiation of BMSCs, an miR-16 mimic was used to up-regulate expression of miR-16. Western blots revealed that upregulation of miR-16 reduced the MgCl_2_-induced levels of osteogenic differentiation makers, ALP, RUNX2, Sp7, OCN, and OPN (Figure 6(a)-6(b)). Moreover, the MgCl_2_-induced increases of ALP activity were repressed (Figure 6(c)), and the high ALP content and mineralized nodule formation induced by MgCl_2_ were suppressed (Figure 6(d)). In order to further demonstrate the effect of miR-16 on osteogenic differentiation of BMSCs, we added miR-16 inhibitor to the medium. We found that the inhibitor was effective in inhibiting the expression of miR-16 (Figure S3A). Moreover, the treatment of miR-16 inhibitor increased the protein levels of ALP, Runx2, Sp7, OCN, and OPN (Figure S3B-C), ALP activity (Figure S3D), and ALP content and mineralized nodules f a complete well was increased during osteogenic differentiation after the treatment of miR-16 inhibitor (Figure S3E). Thus, in BMSCs, miR-16 is involved in the MgCl_2_-induced enhancement of osteogenic differentiation, which may be related to that the inhibition of miR-16 enhances osteogenic differentiation of BMSCs.

### 3.8. miR-16 Is Involved in MgCl_2_-Promoted Osteogenic Differentiation via FGF2 Regulation of the ERK/MAPK Pathway in BMSCs

**Figure 7:**
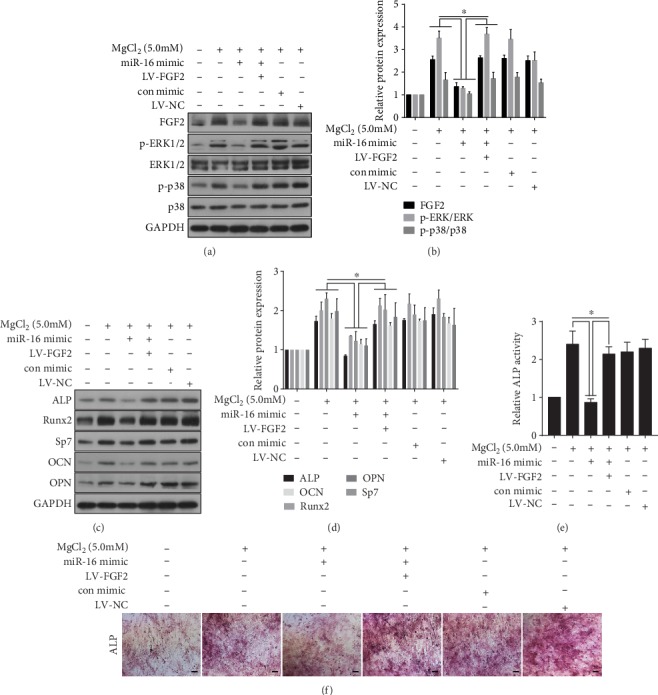
miR-16 is involved in MgCl_2_-promoted osteogenic differentiation via FGF2 regulation of the ERK/MAPK pathway in BMSCs. BMSCs were co-transfected with the miR-16 mimic or with the miR-16 mimic + LV-FGF2 for 24 h, then exposed to 0 or 5.0 mM MgCl_2_ for 48 h. (a) Western blots were performed, and (b) relative protein levels of FGF2, p-ERK1/2, ERK1/2, p-p38, and p38 were determined (mean ± SD, *n* = 3). ^∗^ *p* < 0.05, different from BMSCs treated with MgCl_2_ in the absence of the miR-16 mimic or transfected with LV-FGF2. BMSCs were co-transfected with the miR-16 mimic or with the miR-16 mimic + LV-FGF2 for 24 h, then exposed to 0 or 5.0 mM MgCl_2_ and subjected to osteogenic differentiation for 7 days. (c) Western blots were performed, and (d) relative protein levels of ALP, Runx2, Sp7, OCN, and OPN were determined (mean ± SD, *n* = 3). ^∗^ *p* < 0.05, different from BMSCs treated with MgCl_2_ in the absence of the miR-16 mimic or transfected with LV-FGF2. BMSCs were co-transfected with the miR-16 mimic or with the miR-16 mimic + LV-FGF2 for 24 h, then exposed to 0 or 5.0 mM MgCl_2_ and subjected to osteogenic differentiation for 10 days. (e) ALP activity was detected by ALP assays (mean ± SD, *n* = 3). ^∗^ *p* < 0.05, different from BMSCs (treated with MgCl_2_) in the absence of the miR-16 mimic or transfected with LV-FGF2. BMSCs were co-transfected with the miR-16 mimic or with the miR-16 mimic + LV-FGF2 for 24 h, then exposed to 0 or 5.0 mM MgCl_2_ and subjected to osteogenic differentiation for 14 days. (f) The ALP content and the numbers of mineralization nodules were evaluated by ALP staining (upper) and alizarin red S staining (lower). Scale bar, 100 *μ*m.

**Figure 8:**
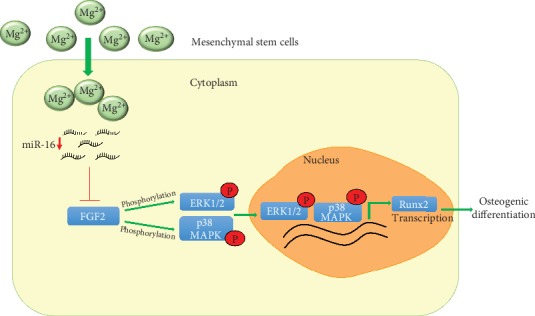
Schematic representation showing the proposed mechanism for osteogenic differentiation caused by MgCl_2_. In BMSCs, MgCl_2_ down-regulates miR-16, followed by upregulation of FGF2, which activates the ERK/MAPK pathway. Activation of ERK and MAPK promotes the transcription of RUNX2, which leads to the osteogenic differentiation of BMSCs.

To confirm that miR-16 is involved in the MgCl_2_-induced enhancement of osteogenic differentiation via FGF2-mediated activation of the ERK/MAPK pathway, BMSCs were co-transfected with an miR-16 mimic and with LV-FGF2. In MgCl_2_-treated BMSCs transfected with the miR-16 mimic, there were lower levels of FGF2, p-p38, and p-ERK, which were restored in these cells co-transfected with the miR-16 mimic and LV-FGF2 (Figure 7(a)-7(b)). In MgCl_2_-treated cells transfected with the miR-16 mimic, there were lower levels of ALP, RUNX2, Sp7, OCN, and OPN (Figure 7(c)-7(d)), ALP activity (Figure 7(e)), and mineralized nodule formation (Figure 8(f)); the levels were restored in MgCl_2_-treated BMSCs co-transfected with the miR-16 mimic and LV-FGF2. These data provide evidence that, for BMSCs, miR-16 is involved in the MgCl_2_-induced promotion of osteogenic differentiation through activation of ERK/MAPK pathway *via* targeting of FGF2.

## 4. Discussion

Approximately 50–60% of Mg in the body is in the skeleton, and dietary Mg deficiency has been implicated as a risk factor for osteoporosis [36]. Administration of 600 mg of Mg per day over 6–12 months to 19 patients demonstrated that the bone mineral density of the calcaneus increased by 11% compared with a 0.7% rise in that of control subjects [37]. In young, growing animals exposed to Mg deficiency, epiphyseal and diaphyseal growth plates are thinned, and there is a decrease in the number and organization of chondrocytes [38]. There are beneficial effects of various Mg alloys on bone formation. Mg-coated prostheses and the balanced combination of Mg^2+^ with calcium and phosphate have demonstrated osteoinductive effects [39, 40]. Supra-physiological concentrations of Mg_2_SO_4_ promote the expression of transcription factors related to *COL10A1* expression [41]. Mg^2+^ is abundant in the skeleton and is essential for bone development in that it allows physiological development, mineralization, and osteogenesis of BMSCs [37]. In the present study, we chose BMSCs to evaluate the mechanism of MgCl_2_ on bone formation.

Surface topography affects the pattern of gene expression of bone-related proteins (OSP, OSN, bone sialoprotein, type I collagen, and ALP) [42]. Moreover, RUNX2, a factor in osteoinduction and transcription, affects osteoblast differentiation by controlling osteoblast-specific gene expression (associated with osteogenesis) of type I collagen, ALP, OSP, and OCN. RUNX2 and Sp7 are osteogenic transcription factors that control bone mineralization and progression in BMSCs and osteoblasts [43, 44]. Enhancing Runx2 and osterix transcriptional activities promotes osteoblastic differentiation and facilitates osteogenesis [44, 45]. In the present study, BMSCs were treated with 2.5 or 5.0 mM MgCl_2_ and subjected to osteogenic differentiation, which revealed increased expression of osteogenic markers (ALP, RUNX2, Sp7, OCN, and OPN). ALP activity and mineralization also increased. These findings are consistent with previous studies [37, 39]. In addition to their effects on bone formation, Mg ions at optimal concentrations enhance the phenotype of chondrocytes [46], and magnesium deficiency elevates the numbers of adipocytes [47]. High concentrations of magnesium inhibit calcification of the extracellular matrix and protect articular cartilage via the Erk/autophagy pathway [27].

In chronic lymphocytic leukemia cells, expression of miR-16 is low due to a deletion in chromosome 13q14, and low levels of miR-16 inhibit apoptosis through targeting *Bcl-2* [48]. miRNA-16–5p is a member of the miR-15 family that includes miR-15–5p, miR-16–5p, miR-195–5p, miR-322–5p, and miR-497–5p, which share the same seed sequence [49–51]. miRNA-16–5p is involved in cell proliferation, apoptosis, and differentiation and in angiogenesis. miR-16 is downregulated in prostate cancers, breast cancers, and hepatocellular carcinomas [52], indicating that it functions as a tumor suppressor and that re-expression of miR-16 in tumors represents a potential approach to cancer therapy. Also, there is little research about the effect of miRNA-16–5p on differentiation, such as differentiation of myoblasts [53] and pre-B cells [54]. Further, little is known about the role of miRNA-16 in the metabolism and differentiation of BMSCs. In the present research, we demonstrated that miRNA-16 expression was reduced in BMSCs treated with MgCl_2_. After over-expression of miR-16 in BMSCs, the levels of miR-16 were elevated, and the osteogenic differentiation of BMSCs promoted by MgCl_2_ was blocked, which confirmed that miR-16 is involved in the regulation of osteogenic differentiation enhanced by MgCl_2._

MSC-mediated tissue repair and regeneration can be achieved by their cell proliferation and differentiation capacities as well as their paracrine effects that provide secreted cytokines and proteinases, such as VEGF, MMPs, TGF-*β*, and basic FGF (bFGF, also known as FGF2), required for angiogenesis and tissue remodeling [55]. Intracellular signaling activation induced by bFGF has been explored for MSCs originating from various tissues. For instance, bFGF-triggered activation of AKT and the ERK pathway promotes differentiation and proliferation of BMSCs [56]. Phosphorylation of the ERK1/2 signaling pathway is a trigger of osteogenic differentiation [57]. Human amnion-derived MSCs (HAMSCs) promote osteogenic and angiogenic differentiation of HASCs, which is mediated by phosphorylation by the ERK1/2 MAPK signaling pathway [58]. FGF2 regulates proliferation of osteogenic precursor cells and Runx2 function, thereby promoting osteoblast differentiation via the ERK1/2 and p38 MAPK pathways [59]. Mg ions cause elevated levels of FGF2 and promote proliferation and osteogenic differentiation of BMSCs [60]. The results of the present study show that, after treatment with MgCl_2,_ the levels of FGF2 were elevated, along with activation of the ERK1/2 and p38 MAPK pathways. To investigate the role of FGF2 in this process, FGF2 was downregulated. In these cells, activation of the ERK1/2 and p38 MAPK pathways was reduced. Simultaneously, the promotion of osteogenic differentiation of BMSCs induced by MgCl_2_ was blocked in cells with loss of function of FGF2. Therefore, the results indicated that FGF2 was essential for regulation of the osteogenic differentiation caused by MgCl_2._

Overexpression of miR-16 inhibits FGF2 expression, and miR-16 blocks proliferation and migration of prostate cancer cells by reducing FGF2 expression [61]. Therefore, we speculated that FGF2 is a target gene of miR-16. We predicted by bioinformatics that miR-16 acts on the 3'-UTR of FGF2 and confirmed, by luciferase reporter gene assays, that miR-16 inhibited the FGF2 luciferase activity of BMSCs. This suggested that miR-16 inhibits the osteogenic differentiation of BMSCs by down-regulation of FGF2. Moreover, down-regulation of miR-16 enhanced the osteogenic differentiation of BMSCs. Further, for BMSCs, overexpression of FGF2 increased activation of the ERK/MAPK pathway and promoted the osteogenic differentiation induced by MgCl_2._ The results help to understand potential biomaterials for orthopedic implants, biodegradable magnesium-containing materials have advantages, including self-degradation and promotion of bone repair.

However, this investigation was a preliminary experiment in vitro to research the role of magnesium ions in bone repair and regeneration and can promote the development of better biomaterials for bone defect repair. Therefore, the weakness of the current study is that it is only based on cell culture system, which may not reflect the in vivo situation. There are still a lot of works to be done for clinical application, such as we have launched animal experiments on the basis of cell culture system to investigate the roles and mechanisms of miR-16/FGF2 axis in magnesium hydroxyapatite coating C/C composite promoting the repair and reconstruction of bone defects.

## 5. Conclusion

In conclusion, our work showed, for BMSCs, a relationship between changes in miR-16 expression, Mg ion concentrations, and osteogenic differentiation and clarified the molecular mechanism involved. Moreover, for BMSCs, MgCl_2_ caused a decrease of miR-16, which up-regulated FGF2, a target of miR-16. FGF2 activated the ERK/MAPK pathway and enhanced the osteogenic differentiation of BMSCs. Thus, in BMSCs exposed to MgCl_2_, miR-16 controls osteogenic differentiation via regulation of FGF2 and the ERK/MAPK pathway (Figure 8).

## Figures and Tables

**Table 1:** Primer sequences used.

GAPDH	5'-GCATCCTGGGCTACACTG-3'
	5'-TGGTCGTTGAGGGCAAT-3'
ALP	5'-GGTCACCAGGGCTGCTTTTA-3'
	5'-GGATCTCGCTCCTGGAAGATG-3'
OCN	5'-CCACGTCTTCACATTTGGTG-3'
	5'-AGACTGCGCCTGGTAGTTGT-3'
Runx2	5'-CATGAGGACCCTCTCTCTGC-3'
	5'-TGGACATGAAGGCTTTGTCA-3'
Sp7	5'-TGTCATGGCGGGTAACGAT-3'
	5'-AAGACGGTTATGGTCAAGGTGAA-3'
OPN	5'-GAGGCAACTGGCTAGGTGG-3'
	5'-CTGGATTAAGGGGAGCAAAGTC-3'
miR-16	5'- TAGCAGCACGTAAATATTGGCG -3'
	5'- TGGTGTCGTGGAGTCG -3'
miR-214	5'- GGGAGAGTTGTC -3'
	5'- TGGTGTCGTGGAGTCG -3'
miR-215	5'- GGGATGACCTATGATTT -3'
	5'- TGGTGTCGTGGAGTCG -3'
miR-192	5'- GGGCTGACCTATGAAT -3'
	5'- TGGTGTCGTGGAGTCG -3'
miR-542	5'- GGGTGTGACAGATTGAT -3'
	5'- TGGTGTCGTGGAGTCG -3'
	5'- TGGTGTCGTGGAGTCG -3'
U6	5'-ACCCTGAGAAATACCCTCACAT-3'
	5'-GACGACTGAGCCCCTGATG-3'

## Data Availability

The data used to support the findings of this study are available from the corresponding author upon request.

## Abbreviations

Mg: Magnesium
MSCs: Mesenchymal stem cells
BMSCs: Bone marrow mesenchymal stem cells
FGF2: Fibroblast growth factor 2
ALP: Alkaline phosphatase
Runx 2: Runt-related transcription factor 2
Sp7: Osterix
OCN: Osteocalcin
OPN: Osteopontin
bFGF: Basic fibroblast growth factor
qRT-PCR: Quantitative reverse-transcriptase polymerase chain reaction.

## Supplementary Materials

Supplementary Materials Figure S1. Cytotoxicity of MgCl2 to BMSCs. Figure S2. MgCl2 promotes increased the ALP content and the numbers of mineralization nodules during the osteogenic differentiation of BMSCs in dose-effect manner. Figure S3. Inhibition of miR-16 promotes the osteogenic differentiation of BMSCs.
