# Supplementary material for: MicroRNA-16, via FGF2 Regulation of the ERK/MAPK Pathway, Is Involved in the Magnesium-Promoted Osteogenic Differentiation of Mesenchymal Stem Cells
Source: Oxid Med Cell Longev. 2020 Apr 27;2020:3894926. doi: 10.1155/2020/3894926 (PMC7201663; doi:10.1155/2020/3894926)
Supplement: Supplementary Materials — Figure S1. Cytotoxicity of MgCl2 to BMSCs. Figure S2. MgCl2 promotes increased the ALP content and the numbers of mineralization nodules during the osteogenic differentiation of BMSCs in dose-effect manner. Figure S3. Inhibition of miR-16 promotes the osteogenic differentiation of BMSCs. [file 3894926.f1.docx]

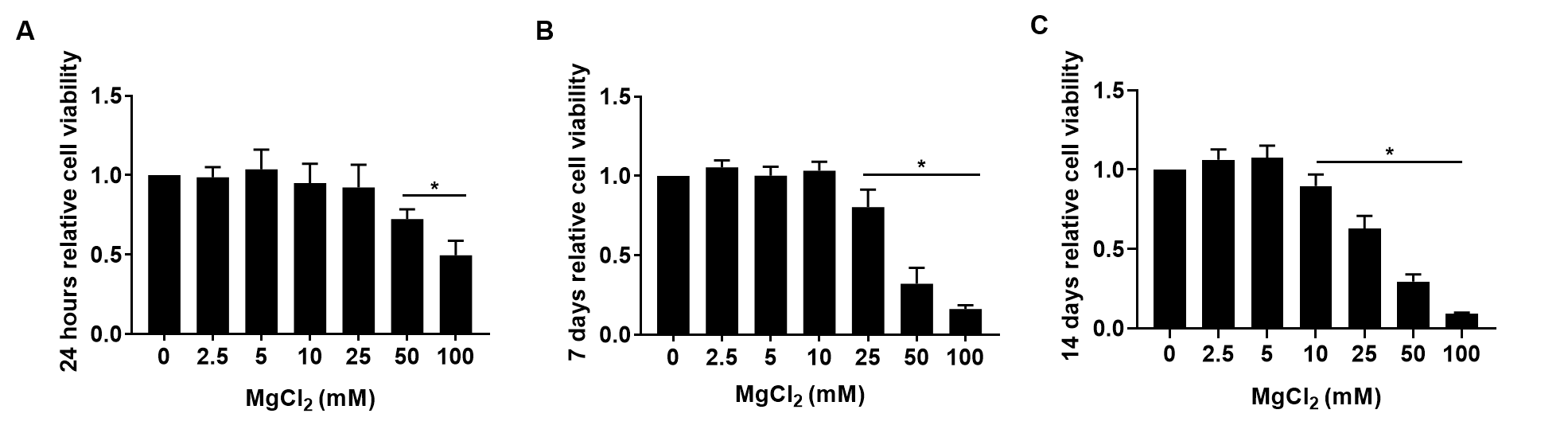


***Figure S1. Cytotoxicity of MgCl_2_ to BMSCs.***

BMSCs were exposed to 0, 2.5, 5, 10, 25, 50, or 100 mM MgCl_2_ for 24 h (A), 7 days (B) or 14 days (C). The viability of BMSCs was assessed by MTT analysis (mean ±SD, n = 5). * *p* < 0.05, different from BMSCs in the absence of MgCl_2_.


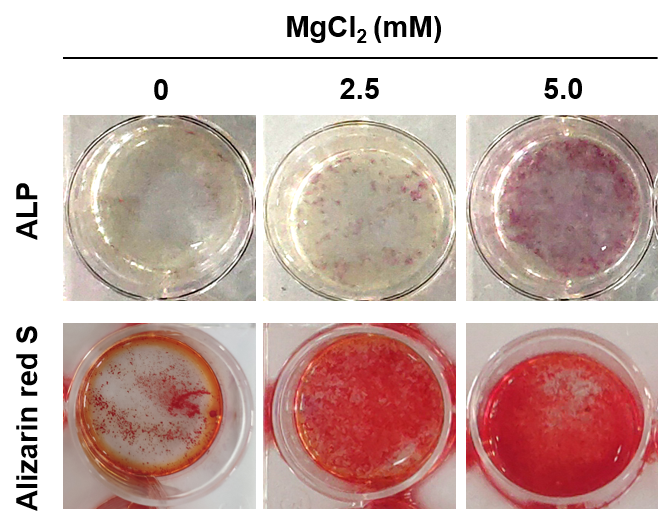


***Figure S2. MgCl_2_ promotes increased the ALP content and the numbers of mineralization nodules during the osteogenic differentiation of BMSCs in dose-effect manner.***

MSCs were exposed to 0, 2.5, or 5.0 mM MgCl_2_ and subjected to osteogenic differentiation for 14 days. The ALP content and the numbers of mineralization nodules were evaluated by ALP staining (upper) and alizarin red S staining (lower).


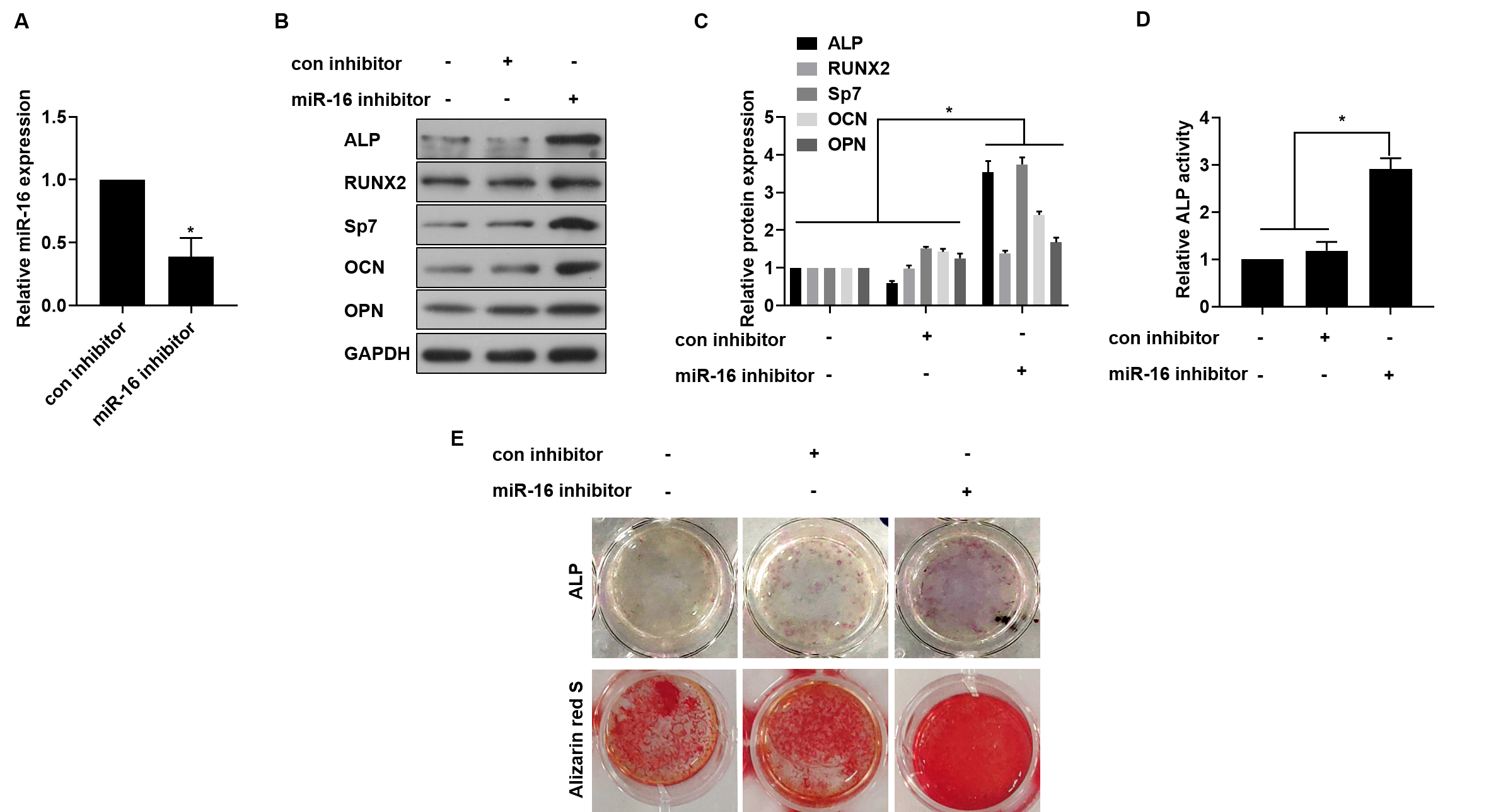


***Figure S3. Inhibition of miR-16 promotes the osteogenic differentiation of BMSCs.***

BMSCs were transfected for 24 h with the miR-16 inhibitor or negative control. (A) The levels of miR-16 were determined by qRT-PCR (mean ± SD, *n* = 3). * *p* < 0.05, different from BMSCs treated with control inhibitor. BMSCs were transfected with miR-16 inhibitor or a negative control and subjected to osteogenic differentiation for 7 days. (B) Western blots were performed, and (C) relative protein levels of ALP, Runx2, Sp7, OCN, and OPN were determined (mean ± SD, *n* = 3). * *p* < 0.05, different from BMSCs treated with or without the miR-16 inhibitor. BMSCs were transfected with miR-16 inhibitor or a negative control and subjected to osteogenic differentiation for 10 days. (D) ALP activity was detected by ALP assays (mean ± SD, *n* = 3). * *p* < 0.05, different from BMSCs treated with or without the miR-16 inhibitor. BMSCs were transfected with miR-16 inhibitor or a negative control and subjected to osteogenic differentiation for 14 days. (E) The ALP content and the numbers of mineralization nodules were evaluated by ALP staining (upper) and alizarin red S staining (lower).
